# Atherogenic Index in Type 2 Diabetes and Its Relationship with Chronic Microvascular Complications

**DOI:** 10.1155/2018/1765835

**Published:** 2018-11-29

**Authors:** Zhen Li, Qi Huang, Li Sun, Tengfei Bao, Zhe Dai

**Affiliations:** Department of Endocrinology, Zhongnan Hospital of Wuhan University, Wuhan 430071, China

## Abstract

**Objective:**

This study was designed to investigate risk factors related to atherogenic index of plasma (AIP), as well as the relationship between AIP and chronic microvascular complications in patients with type 2 diabetes (T2DM).

**Methods:**

This study included 2523 patients with T2DM who had not been treated with lipid-lowering drugs and were admitted to the Department of Endocrinology at Zhongnan Hospital, Wuhan University, during the period from January 2015 to February 2018. Anthropometric indicators were measured after overnight fasting. Low-density lipoprotein cholesterol (LDL-C), high-density lipoprotein cholesterol (HDL-C), total cholesterol (TC), and triglyceride (TG) were detected by enzymatic analysis. Standard 75 g oral glucose tolerance testing was performed to measure 0 and 2 hr plasma levels of glucose and insulin. Insulin sensitivity was assessed with HOMA-IR.

**Results:**

Increase in AIP was associated with an increased risk for hypertension (*P* < 0.05), HbA_1c_ (*P* < 0.05), HOMA-IR (*P* < 0.05), UA (*P* < 0.05), and decreased eGFR levels (*P* < 0.05). Furthermore, AIP values directly correlated with BMI (*r* = 0.182, *P* < 0.001), waist circumference (*r* = 0.129, *P* < 0.001), blood glucose index (FBG (*r* = 0.153, *P* < 0.001), PPBG (*r* = 0.117, *P* < 0.001), and HbA_1c_ (*r* = 0.074, *P* < 0.001)), insulin resistance (HOMA-IR; *r* = 0.112, *P* < 0.001), and uric acid (UA, *r* = 0.177, *P* < 0.001). Multiple logistic regression analysis showed that waist circumference, HOMA-IR, FBG, systolic blood pressure, and UA were independent risk factors for AIP (all *P* < 0.05). The prevalence of diabetic neuropathy and metabolic syndrome was significantly higher among patients with higher AIP.

**Conclusion:**

AIP represents a clinically convenient indicator for the detection of T2DM with high risk of complications and associated diseases and thus is a good predictor and indicator for follow-up monitoring in the treatment of patients with high-risk type 2 diabetes.

## 1. Introduction

Type 2 diabetes is a major risk factor for cardiovascular disease (CVD). The risk of CV mortality in patients with type 2 diabetes (T2DM) is 2-4 times that observed in individuals without diabetes [1]. CVD accounts for about 70% death casualty of patients with T2DM [2]. Early assessment and control of CV risk factors in patients with T2DM has a positive effect on reducing the risk of CVD and death in patients and improving the prognosis of patients. The atherogenic index of plasma (AIP) is a good predictor of the risk of atherosclerosis and coronary heart disease [3, 4]. The AIP is related to the size of antiatherosclerotic lipoprotein particles. This measure thus reflects the balance between protective and atherogenic lipoproteins [5].

Some studies have suggested that the visceral fat area in patients with T2DM is associated with AIP [6]. High AIP may also increase the risk of T2DM [7]. Patients with type 2 diabetes with metabolic syndrome also have higher AIP than T2DM patients without metabolic syndrome [8]. Treatment with pioglitazone may help to reduce the AIP in diabetics and animal models [9, 10]. However, at present, we do not fully understand the relationship between AIP-related risk factors and microvascular complications in patients with T2DM. Therefore, in a large sample cross-sectional study, AIP-related risk factors and their relationship with arterial vascular plaques in patients with T2DM without lipid-lowering drugs were investigated. The possibility of AIP as a predictor and follow-up monitoring factor in assuming patients with high-risk type 2 diabetes was explored.

## 2. Materials and Methods

### 2.1. Subjects

A total of 2523 patients with T2DM without lipid-lowering drugs who were admitted to the Department of Endocrinology of Wuhan University from January 2015 to February 2018 were included. The average age was 53.9 ± 12.7 years, including 1353 males and 1170 females. Exclusion criteria included the presence of acute disease, severe chronic disease, liver or kidney dysfunction, pregnancy, type 1 diabetes, and secondary diabetes. This study was approved by the Ethics Committee of Zhongnan Hospital of Wuhan University.

### 2.2. Anthropometric Indicators

All patients measured height, weight, and waist circumference after 8 h fasting and calculated body mass index (BMI) = body weight (kg)/height (m)^2^. The standard blood pressure was measured three times, and mean blood pressure was calculated. The diagnostic criteria for hypertension were those proposed by the China Guidelines for the Prevention and Treatment of Hypertension published in 2011.

### 2.3. Biochemical Indicators Testing

Low-density lipoprotein cholesterol (LDL-C), high-density lipoprotein cholesterol (HDL-C), total cholesterol (TC), and triglyceride (TG) were detected after 10 hours overnight fasting. LDL-C (mmol/L) was measured using surfactant-based homogeneous assay, TC (mmol/L) was measured using enzymatic method, HDL-C (mmol/L) was measured using peroxidase method, and TG (mmol/L) was measured using glycerol-3-phosphate oxidase (GPO) and peroxidase (POD) method.

The 75 g glucose tolerance test was underwent for the detection of plasma glucose and insulin levels at fasting and 2 hr postloading. All biochemical tests were performed on Beckman AU5400 autoanalyzer. Hemoglobin A1c (HbA1c) was measured using ion-exchange high-performance liquid chromatography (HPLC) method. Creatinine (Scr, *μ*mol/L) was measured using enzymatic method. Serum uric acid (UA, *μ*mol/L) was measured using urease-peroxidase-based method. Blood urea nitrogen (BUN, mmol/L) was measured using enzymatic method. The MDRD formula available on the website for the National Institutes of Health was used to calculate estimated glomerular filtration rate (eGFR). eGFR (mL/min∙1.73 m^2^) = 186 × (Scr) – 1.154 × (age) – 0.203 × (0.742 female).

### 2.4. Identification of Chronic Microvascular Complications

Diabetic retinopathy (DR), diabetic nephropathy, and diabetic peripheral neuropathy were diagnosed according to the management guidelines for T2DM published by the Chinese government in 2017. The criteria for each complication are described in detail below.

For the diagnosis of diabetic retinopathy (DR), all patients included in the study underwent fundus photography and fluorescein fundus angiography (TRC-50DX, Topcon, Tokyo, Japan). Both eyes in each patient were examined. DR was diagnosed by an ophthalmologist. DR was classified as nonproliferative or proliferative. Nonproliferative DR showed one or more of the following symptoms: microaneurysm, hemorrhage, exudates, or microvascular abnormalities; proliferative DR showed the generation of new vessels and fibrosis.

For the diagnosis of diabetic nephropathy (DN), 24 hr urine samples were collected in order to measure the urinary microalbumin (UMA) levels. Normoalbuminuria was defined as 24 hr UMA < 30 mg/24 hr, microalbuminuria as 24 hr UMA = 30-299 mg/24 hr, and macroalbuminuria as 24 hr UMA ≥ 300 mg/24 hr. T2DM patients with microalbuminuria or macroalbuminuria were diagnosed as having DN. eGFR was calculated using the simplified Modification of Diet in Renal Disease equation.

Briefly, the criteria for diabetic peripheral neuropathy (DPN) were as follows: (1) confirmed T2DM; (2) decreased sensation and positive neuropathic sensory symptoms (including pricking, burning, stabbing, and aching pain) in the toes, feet, or legs; (3) decreased distal sensation, unequivocally decreased, or absent ankle reflexes; and (4) abnormal motor and sensory nerve conduction.

### 2.5. Definition of Metabolic Syndrome

Metabolic syndrome was determined by the definition proposed by the China Diabetes Society (CDS) [11], which requires three or more abnormalities of the following criteria: (1) overweight or obesity (BMI ≥ 25.0 kg/m^2^), (2) dyslipidemia (TG ≥ 1.70 mmol/L and/or low HDL-cholesterol (<0.9 mmol/L in men and <1.0 mmol/L in women)), (3) hypertension (SBP ≥ 140 mmHg, DBP ≥ 90 mmHg, or on antihypertensive medication), and (4) hyperglycemia (FBG ≥ 6.1 mmol/L and/or 2-hour postprandial plasma glucose (PPBG) ≥ 7.8 mmol/L or under treatment for diabetes).

### 2.6. Calculation of AIP, Islet Cell Function, and Insulin Sensitivity

AIP was calculated as log (TG/HDL–C). Islet cell function was evaluated using the following index: HOMA-*β* = ((20 × FINS (*μ*U/mL))/(FBS (mmol/L)-3.5)). Insulin sensitivity was evaluated using the following index: HOMA-IR = FINS (*μ*U/mL) × FBS (mmol/L)/22.5.

### 2.7. Statistics

Statistical analysis was performed using SPSS 16.0 statistical software. Nonnormally distributed data needs to be analyzed after being converted to normal distribution data. Data are expressed as ±sd or percentages. Measurement data were analyzed statistically with independent-sample *t*-test or analysis of variance. The Kruskal-Wallis H-test was used when the variance was not uniform. Count data using *χ*^2^ test, when *T* < 1, using the exact probability method, continuous variables using multiple logistic regression analysis to explore the relevant risk factors of AIP.

## 3. Results

### 3.1. Baseline Data

AIP for the overall patient population was 0.11 ± 0.31, then was submitted to tripartite analysis. The tertiles were AIP Q1 (<−0.13), AIP Q2 (−0.13–0.23), and AIP Q3 (≥0.23). According to the results (Table 1), compared with the AIP Q1 group, patients with AIP Q2 and/or AIPQ3 had higher proportion of hypertension (*P* < 0.05), BMI (*P* < 0.05), and waist circumference (*P* < 0.05). The hip circumference (*P* < 0.05) and waist-to-hip ratio (*P* < 0.05) increased significantly, FBS (*P* < 0.05), PPBS (*P* < 0.05), HbA_1c_ (*P* < 0.05) levels, HOMA-IR levels, TG levels were significantly increased (*P* < 0.05), and HDL-C levels were significantly decreased (*P* < 0.05). Although levels of FINS (*P* < 0.05) and PINS (*P* < 0.05) increased, there was no significant difference in HOMA-*β* between groups (*P* > 0.05). Patients with AIP Q2 and/or AIPQ3 had significantly higher UA levels (*P* < 0.05) than those in AIP Q1 group and decreased eGFR levels (*P* < 0.05).

### 3.2. Correlation Analysis of Difference Indexes of AIP

**Figure 1:**
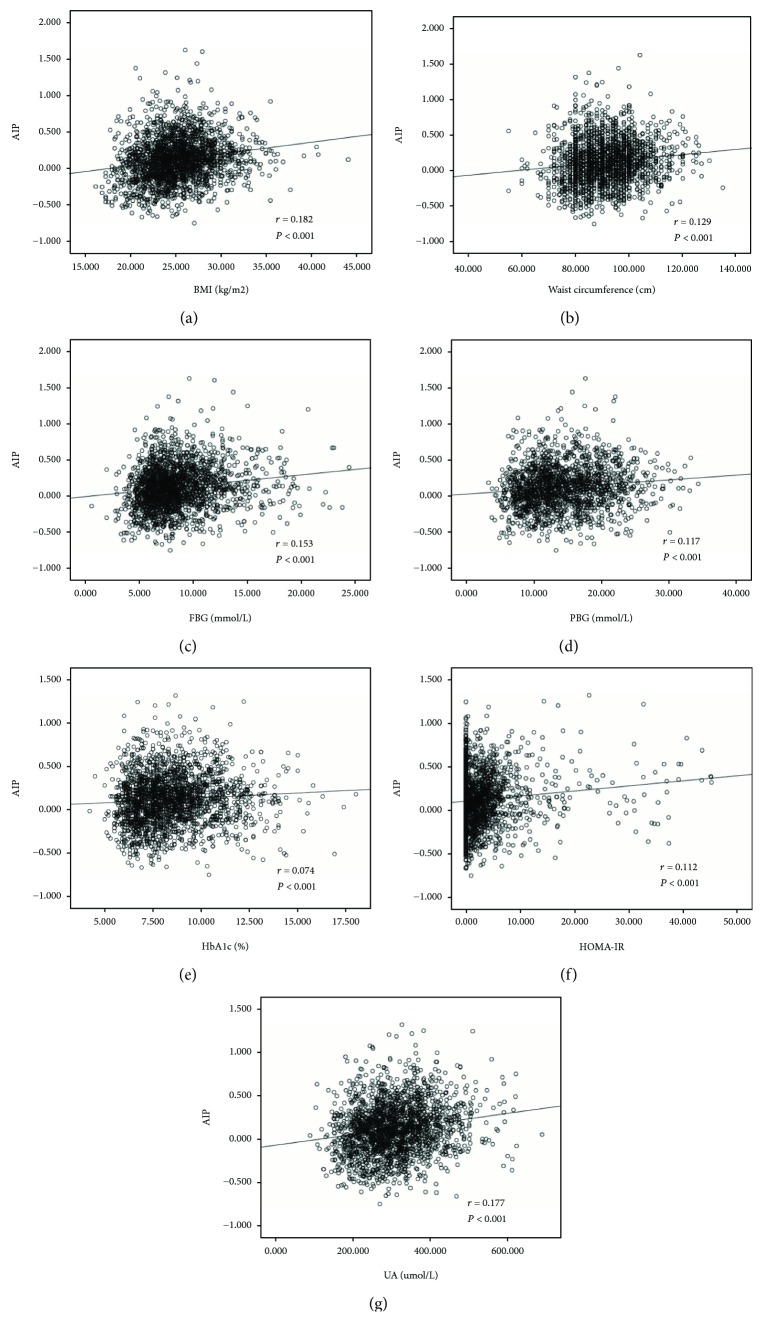
Correlation analysis for AIP and BMI (a), waist circumference (b), fasting glucose (c), postprandial glucose (d), HbA_1c_ (e), HOMA-IR (f), and uric acid (g).

Based on the data analysis of different AIP tertile populations, we further analyzed these disparate indicators. AIP was significantly correlated with BMI (*r* = 0.182, *P* < 0.001), waist circumference (*r* = 0.129, *P* < 0.001), blood glucose index (FBG; *r* = 0.153, *P* < 0.001), PPBG (*r* = 0.117, *P* < 0.001), HbA_1c_ (*r* = 0.074, *P* < 0.001), insulin resistance-related indicators (HOMA-IR; *r* = 0.112, *P* < 0.001), and uric acid (UA, *r* = 0.177, *P* < 0.001). No significant correlation between AIP and islet function was observed (FINS; *P* = 0.28; PINS, *P* = 0.51; HOMA-*β*, *P* = 0.36) (Figure 1).

### 3.3. Logistic Multiple Regression Analysis of AIP

To further analyze the risk factors of AIP in patients with T2DM, we used AIP as an independent variable; gender, DM family history, BMI, waist circumference, systolic blood pressure, diastolic blood pressure, FBG, PPBG, HbA_1c_, FINS, PINS, HOMA-IR, HOMA-*β*, BUN, Cr, UA, and eGFR were used as the dependent variables for multiple logistic regression analysis. Analysis showed that waist circumference, HOMA-IR, FBG, systolic blood pressure, and UA were independent risk factors for AIP (all *P* < 0.05) (Table 2).

### 3.4. Correlation between AIP and Chronic Microvascular Complications

**Figure 2:**
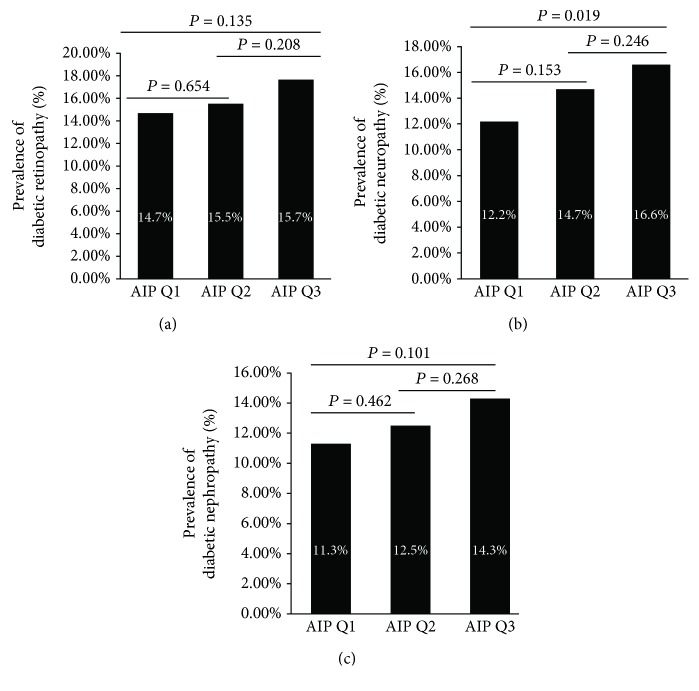
Prevalence of diabetic retinopathy (a), diabetic neuropathy (b), and diabetic nephropathy (c) across groups.

In order to analyze the correlation between AIP and chronic microvascular complications, we compared the prevalence of different complications among different AIPs (Figure 2). DR and DN showed similar prevalence among groups. The prevalence of DPN is significantly higher in AIP Q3 group compared to AIP Q1 group, but not statistically significant between AIP Q1 and AIP Q2 groups.

### 3.5. Correlation between AIP and Metabolic Syndrome

**Figure 3:**
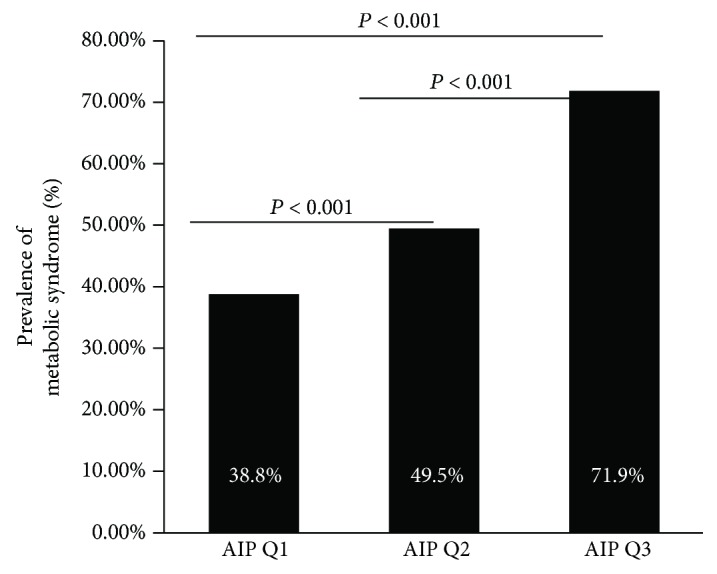
Prevalence of metabolic syndrome across groups.

In order to analyze the correlation between AIP and metabolic syndrome (MS), we compared the prevalence of metabolic syndrome among different AIPs (Figure 3). The prevalence of metabolic syndrome is significantly higher in AIP Q3 and AIP Q2 groups compared to AIP Q1 group.

## 4. Discussion

AIP is considered to be a good predictor of atherosclerosis [3] and a highly sensitive predictor of risk for CVD. AIP values show substantial agreement with the results of coronary angiography [5] and are used to predict acute coronary events [12] and prognosis in patients with acute myocardial infarction [13]. AIP is superior to other traditional assessment indexes (e.g., cardiogenic risk ratio and atherogenic coefficient) in assessing risk for CV events [14]. AIP is also considered to predict risk for T2DM [7].

Population studies have shown that AIP is associated with waist circumference [15], waist-to-hip ratio [16], BMI [15–17], physical activity [15], age [16], blood pressure [17], and fasting blood glucose [17]. In our study, AIP was mainly related to body weight and body fat correlation index (such as BMI, waist circumference, and waist-hip ratio), blood glucose correlation index (FBG, PPBG, and HbA_1c_), and insulin resistance index (HOMA-IR). Individuals in the group with higher AIP were at an increased risk for hypertension and atherosclerotic plaques. Logistic multiple regression analysis showed that systolic blood pressure, waist circumference, fasting blood glucose, and HOMA-IR were independent risk factors for AIP. The risk of CV and cerebrovascular diseases in patients with T2DM is increased, and the rate of death from disability is high. Risk factors for more aggregation in patients with T2DM include insulin resistance, central obesity, elevated blood pressure, and elevated total triglycerides. The underlying mechanisms include increased oxidative stress, increased inflammation, or endothelial cell dysfunction in association with low levels of HDL cholesterol. The risk factors associated with increased AIP are closely related to those for CVD and cerebrovascular disease in patients with T2DM. Compared with risk factors associated with CVD and cerebrovascular disease, those associated with increased AIP are more conducive to monitoring and follow-up.

The relationship between AIP and UA has been addressed in previous studies. Previous reports found a significantly positive correlation between AIP and UA in patients with diabetes [18], general population [19–21] in different countries, and postmenopausal women [17]. AIP may also be used to predict hyperuricemia [22]. According to the results presented above, individuals with higher AIP also had higher UA levels. Furthermore, a significantly positive correlation between AIP and UA was identified, with UA as a risk factor for AIP, which was also shown in previous study [20]. Several studies have shown that serum UA levels are associated with CVD [23–25], obesity [26, 27], dyslipidemia [28], hypertension [29, 30], and impaired glucose metabolism [31, 32]. Others have reported an association between UA levels and inflammation [33–36] and endothelial dysfunction [35, 37–40]. High serum UA is thought to contribute to numerous chronic metabolic diseases, including diabetes and coronary heart disease [41]. The close relationship of UA to metabolic diseases might be directly related to the effect on endothelial dysfunction, oxidative stress, and inflammation or indirectly related to several metabolic syndrome risk factors. This might be helpful to explain the relationship between AIP and UA.

The relationship between AIP and diabetic microvascular complications has not previously been fully elucidated. Previous studies have indicated that patients with T2DM and increased AIP are at greater risk for microalbuminuria and that AIP is an early predictor of DN [42, 43]. do Socorro Souza e Silva Moura et al. showed that AIP is positively correlated with microalbuminuria in patients with hypertension [44]. Akdoğan et al. showed no difference in AIP between patients with T2DM with retinopathy, compared with patients with T2DM without retinopathy [45]. However, Miric et al. demonstrated that AIP was higher in patients with T2DM with neuropathy, compared to patients with T2DM without neuropathy [46]. The results presented in this study indicate increased risk for microvascular complications in patients with higher AIP. However, only the difference in prevalence of DN was found to be significant.

Compared to microvascular complications, the relationship of AIP to metabolic syndrome shows more consistency across studies. Previous studies show that population with MS had higher level of AIP [8, 47–50]. In addition, higher level of AIP is related to higher risk of MS [51]; prolonged exercise can help to decrease the risk for MS and AIP level [52]. This study, together with other studies, indicates increased incidence of MS in T2DM patients with higher AIP. It also suggested that AIP is a good index to evaluate risk factors of CVD.

AIP and associated risk factors may be improved through management. Measures found to be effective include dietary modifications [53], aerobic exercise [54], and supplementation with EPA [55]. This study added to the store of accumulated knowledge by identifying AIP as a clinically convenient index for detection and positing an association between AIP and risk factors for CVD and cerebrovascular disease. AIP may be used as an index for monitoring patients during follow-up. By improving metabolic indicators such as blood glucose and blood lipids, as well as providing guidance related to diet and exercise, AIP may be reduced to a level that indicates low risk. These efforts should aid in the implementation of clinical programs for diagnosis and treatment.

This study had certain limitations. The cross-sectional nature of this study precluded any determination of the causal relationship between AIP and risk factors for CVD and cerebrovascular disease. The value of AIP in clinical practice needs to be further confirmed by additional prospective follow-up studies and basic research.

## 5. Conclusions

Our study indicate that AIP represents a clinically convenient indicator for detection of T2DM with high risk for complications and associated diseases, and thus is a good predictor and indicator for follow-up monitoring in the treatment of patients with high-risk type 2 diabetes.

## Figures and Tables

**Table 1:** Baseline data analysis across groups.

	AIP Q1	AIP Q2	AIP Q3
Range	< −0.13	−0.13–0.23	≥ 0.23
Cases	592	1076	855
Sex (M/F)	329/263	622/454	402/453
Hypertension (Y/N)	293/299	452/624	306/549
DM family history (Y/N)	169/423	272/804	229/626
Body weight (kg)	64.50 ± 10.68	68.5 ± 11.36	69.55 ± 10.09
BMI (kg/m^2^)	23.90 ± 4.39	25.30 ± 3.43^∗^	25.79 ± 3.27^∗^
Waist circumference (cm)	89.39 ± 30.67	91.49 ± 11.53	92.77 ± 13.86^∗^
Hip circumference (cm)	95.84 ± 11.17	97.29 ± 11.22	99.32 ± 9.37^∗^
WHR	0.92 ± 0.08	0.93 ± 0.10^∗^	0.94 ± 0.09^∗^
Systolic pressure (mmHg)	142.68 ± 12.96	139.17 ± 11.72	137.26 ± 11.29^∗^
Diastolic pressure (mmHg)	82.19 ± 10.22	79.16 ± 9.47^∗^	75.28 ± 8.27^∗^
FBS (mmol/L)	7.96 ± 3.12	8.52 ± 3.05	9.17 ± 3.24^∗^
PPBS (mmol/L)	13.41 ± 5.24	14.31 ± 5.46	15.30 ± 5.42
HbA_1c_	8.16 ± 2.01	8.2 ± 1.95	8.71 ± 1.82^∗^
FINS (*μ*U/mL)	15.7 ± 5.90	18.06 ± 6.17^∗^	20.19 ± 11.22^∗^
PINS (*μ*U/mL)	53.47 ± 10.33	55.09 ± 15.25	63.52 ± 12.75^∗^
HOMA-IR	2.42 ± 0.53	3.47 ± 0.81^∗^	4.00 ± 0.82^∗^
HOMA-*β*	61.80 ± 15.44	65.45 ± 13.35	66.59 ± 15.20
BUN (mmol/L)	6.21 ± 2.84	5.96 ± 2.33	6.24 ± 2.28
Cr (*μ*mol/L)	74.47 ± 15.04	74.83 ± 13.06	77.85 ± 17.71
UA (*μ*mol/L)	288.50 ± 44.81	308.71 ± 46.86	331.76 ± 41.35^∗^
eGFR (mL/min∙1.73 m^2^)	93.99 ± 28.66	93.52 ± 31.44	92.84 ± 33.07^∗^
TG (mmol/L)	0.81 ± 0.27	1.35 ± 0.44^∗^	2.84 ± 0.98^∗^
HDL-C (mmol/L)	1.58 ± 0.53	1.17 ± 0.41^∗^	0.98 ± 0.22^∗^

M: male; F: female; DM: diabetes mellitus; BMI: body mass index; WHR: waist-to-hip ratio; FBS: fasting blood glucose; PPBG: postprandial blood glucose; HbA1c: hemoglobin A1c; FINS: fasting insulin; PINS: postprandial insulin; UA: uric acid; BUN: blood urea nitrogen; Cr: creatine; eGFR: estimated glomerular filtration rate; TG: triglyceride; HDL-C: high-density lipoprotein cholesterol. ^∗^Compared with AIP Q1 group, *P* < 0.05.

**Table 2:** Multiple logistic analysis of factors associated with AIP.

	*β*	SE (*β*)	*T* value	*P* value
Intercept	2.8295	0.5288	6.72	<0.01
Waist circumference	0.2399	0.0024	4.07	<0.01
HOMA-IR	0.2308	0.0073	3.25	<0.01
FBG	0.1977	0.0927	2.38	0.03
Systolic pressure	0.1879	0.0838	2.76	0.01
UA	0.2215	0.0916	2.19	0.01

## Data Availability

The data used to support the findings of this study are available from the corresponding author upon request.

## Abbreviations

AIP: Atherogenic index of plasma
T2DM: Type 2 diabetes mellitus
CVD: Cardiovascular disease
LDL-C: Low-density lipoprotein cholesterol
HDL-C: High-density lipoprotein cholesterol
TC: Total cholesterol
TG: Triglyceride
UA: Uric acid
eGFR: Estimated glomerular filtration rate
FBG: Fasting blood glucose
PPBG: Postprandial blood glucose
FINS: Fasting insulin
BUN: Blood urea nitrogen
BMI: Body mass index
GPO: Glycerol-3-phosphate oxidase
HbA1c: Hemoglobin A1c
SCr: Serum creatinine
DR: Diabetic retinopathy
DN: Diabetic nephropathy
DPN: Diabetic peripheral neuropathy
CDS: China Diabetes Society
SBP: Systolic blood pressure
DBP: Diastolic blood pressure
EPA: Eicosapentaenoic acid.
